# Effects of Slag-Based Silicon Fertilizer on Rice Growth and Brown-Spot Resistance

**DOI:** 10.1371/journal.pone.0102681

**Published:** 2014-07-18

**Authors:** Dongfeng Ning, Alin Song, Fenliang Fan, Zhaojun Li, Yongchao Liang

**Affiliations:** 1 Ministry of Agriculture Key Laboratory of Crop Nutrition and Fertilization, Institute of Agricultural Resources and Regional Planning, Chinese Academy of Agricultural Sciences, Beijing, China; 2 Ministry of Education Key Laboratory of Environment Remediation and Ecological Health, College of Environmental and Resource Sciences, Zhejiang University, Hangzhou, China; Zhejiang University, China

## Abstract

It is well documented that slag-based silicon fertilizers have beneficial effects on the growth and disease resistance of rice. However, their effects vary greatly with sources of slag and are closely related to availability of silicon (Si) in these materials. To date, few researches have been done to compare the differences in plant performance and disease resistance between different slag-based silicon fertilizers applied at the same rate of plant-available Si. In the present study both steel and iron slags were chosen to investigate their effects on rice growth and disease resistance under greenhouse conditions. Both scanning electron microscopy (SEM) and transmission electron microscopy (TEM) were used to examine the effects of slags on ultrastructural changes in leaves of rice naturally infected by *Bipolaris oryaze*, the causal agent of brown spot. The results showed that both slag-based Si fertilizers tested significantly increased rice growth and yield, but decreased brown spot incidence, with steel slag showing a stronger effect than iron slag. The results of SEM analysis showed that application of slags led to more pronounced cell silicification in rice leaves, more silica cells, and more pronounced and larger papilla as well. The results of TEM analysis showed that mesophyll cells of slag-untreated rice leaf were disorganized, with colonization of the fungus (*Bipolaris oryzae*), including chloroplast degradation and cell wall alterations. The application of slag maintained mesophyll cells relatively intact and increased the thickness of silicon layer. It can be concluded that applying slag-based fertilizer to Si-deficient paddy soil is necessary for improving both rice productivity and brown spot resistance. The immobile silicon deposited in host cell walls and papillae sites is the first physical barrier for fungal penetration, while the soluble Si in the cytoplasm enhances physiological or induced resistance to fungal colonization.

## Introduction

Silicon (Si) is the second most abundant element in soils [1], [2]. Although Si has not been proven to be an essential element for plant growth and development, its beneficial roles in stimulating plant growth, grain yield and resistance to abiotic (metal toxicity, salt and drought stress, nutrient imbalance, extreme temperature) and biotic stress (plant diseases and insect pests) have been well documented [3]–[7].

Rice (*Oryza sativa* L.) is the second most widely grown crop in the world, and the major staple food for more than half of the world's population [8], [9]. Rice is also a typical Si hyper-accumulating plant species, containing Si up to 10% in shoots on a dry weight basis [2]. Rice roots take up Si in the form of silicic acid (H_4_SiO_4_) from the soil solution [10]. In tropical and subtropical areas, because of heavy desilication-aluminization arising from high temperature and rainfall, plant- available Si is low in these highly-weathered soils [11]. In addition, repeated mono-cropping with rice may greatly decrease plant-available Si in soil. It is estimated that producing a total rice grain yield of 5000 kg ha^−1^ will remove Si at 230–470 kg ha^−1^ from the soil [5], and Si may then become a yield-limiting element for rice production [12]–[14]. Therefore, it maybe is necessary to provide exogenous Si fertilizer for an economic and sustainable rice production system [15]–[18].

Brown spot caused by the fungus (*Bipolaris oryzae*) is one of the most devastating and prevalent diseases of rice. Brown spot may cause significant yield losses [19], [20]. The major method to control brown spot in agriculture is through application of fungicides [19]. However, there is a need to explore more eco-friendly management practices in consideration of the public's concerns with health and environmental issues. The physiological condition of rice plant, which is strongly influenced by soil conditions, particularly soil nutrient status (e.g. potassium, calcium, magnesium, manganese, iron, and silicon etc.), is one of the main factors governing brown spot severity [19], [21]. Some authors suggest that application of Si fertilizer to rice fields is an alternative approach to control brown spot, especially in soils where plant-available Si is very low [22]–[24].

Steel slags or iron slags are byproducts of steel or iron industries, which account for 15–20% of total steel production. Large amounts of slag are produced in China annually [25]. Slags are not merely metallurgical wastes, but they have been successfully used in agriculture in many developed counties [26], [27]. In contrast, only 10% of the total slag is recycled in China [28]. Slags contain sufficient amounts of Si (10–28%); therefore, they may potentially be used as a Si fertilizer source. Application of such kind of Si fertilizer has been shown to improve degraded paddy soils, as well as rice growth and disease resistance [2], [29]–[34]. So, slag applied to paddy rice fields as Si fertilizer is beneficial not only for rice health and growth, but also from economic and environmental perspectives. However, variation exists in the ore and coke, as well as in the cooling process; consequently, the composition and property of slags may vary widely [35]. Therefore, plant-available Si content and Si availability in slags vary widely too [36]. Previous studies have demonstrated positive effects of wollastonite or calcium silicate as Si resource on rice growth and disease resistance [30], [32]. However, there are only a few reports to compare the agronomic benefits of different sources of slag used in rice.

The objective of this study was to assess the effects of steel slag and iron slag applied at the same rates of plant-available Si on rice growth and brown spot development in rice and to investigate the relationship between Si-mediated ultrastructural changes and brown spot disease infection in rice.

## Materials and Methods

### Soil and plant material preparation

The soil used was sampled from Qionghai, Hainan province of South China (N 19°09′16.2″, E 110°17′35.3″) (no specific permissions were required for soil sampling in this location and the field in this study did not involve endangered or protected species). It was a latosol derived from basalt with a plant-available Si concentration of 41.8 mg kg^−1^ (extracted by 0.025 M citric acid) and a pH value of 5.16. The soil was air-dried and sieved (2.0 mm). The rice variety tested is a hybrid (*Oryza sativa* L. cv. Fengyuanyou 299), characterized by its mid-late maturity. Seeds were sterilized with 10% (v/v) H_2_O_2_ for 15 min, rinsed with distilled water, soaked in water for 24 hours, and then transferred into culture dishes for germination at 25°C in the dark. Two days later, the germinated seeds were placed on a float tray (10×15 cm) in a controlled environment with a day/night temperature of 25°C (12 h): 25°C (12 h).

### Experimental design

A pot experiment factorically arranged in a 2×4 randomized, complete block design was conducted with three replicates per treatment, giving a total of 24 pots. The entire experiment was duplicated. Two different Si fertilizers were chosen for the pot experiment. One was derived from air-cooling steel slag, with HCl-soluble Si content of 7.61%, referred to as H, and the other was based on water-cooling iron slag, with HCl-soluble Si content of 9.35%, referred to as Q. The main chemical properties of the two slags are presented in Table 1. Four Si treatments with three replicates each were established. The rate of Si applied, equivalent to 0.5 M HCl-soluble Si, was 0 (Si_0_), 187 (Si_1_), 560 (Si_2_) and 935 (Si_3_) mg Si kg^−1^. The Si fertilizer was thoroughly mixed with soil prior to potting. Basal fertilizers supplied were 0.2 g N kg^−1^ as urea, 52 mg P kg^−1^ as potassium dihydrogen phosphate, and 84 mg K kg^−1^ as potassium sulfate. Each plastic pot was filled with 5 kg of air-dried and sieved (2.0 mm) soil. Uniform seedlings with three leaves fully expanded were transplanted at two seedlings per pot. During the rice growing period, distilled water was applied to maintain a 2-cm water layer but no pesticides were applied.

**Table 1:** The main chemical characteristics of two slag-based silicon fertilizers tested in the present study (%).

Si fertilizer	CaO	SiO_2_	MgO	Al_2_O_3_	Fe_2_O_3_	MnO	TiO_2_
Q	43.6	26.9	8.1	10.9	3.1	0.9	1.2
H	50.9	21.0	7.7	6.0	5.0	1.5	0.6

### Plant sampling

Rice plants were harvested at maturity, and separated into stem, leaf, and grain, and then washed thoroughly with distilled water. The dry weight of these tissues was recorded after being oven-dried at 75°C till a constant weight. These tissues were then ground to pass through a 0.5-mm sieve for Si analysis.

### Disease index survey

Rice leaves were naturally infected by *Bipolaris oryaze*, the causal agent of brown spot at the joining stage. Disease severity, based on the percentage of infected leaf surface area and the percentage of infected leaves per pot, was determined two weeks after infection. In this study, disease severity (DS) was classified into nine grades based on the following: DS0  =  healthy plants, DS1≤1%, DS3 = 2–5%, DS5 = 6–15%, DS7 = 16–25% and DS9≥25%. Disease index (%)  =  [∑(S*n_s_)/(9*Ns)] *100. Where S is the severity value, n_s_ is the number of infected leaves with a severity of S and Ns is the number of leaves evaluated [37].

### Scanning electron microscopy

The deposition of Si in the leaf was observed using scanning electron microscopy (SEM). Since similar slag effects on plant growth and brown spot resistance were observed for both Si sources, only leaf samples of rice plants treated with slag H were collected for microscopic examination. At the anthesis stage, fresh specimens of the top-second leaf of rice plants grown without slag (control) or with slag (H) applied at a rate of 935 mg plant-available Si per kg soil were randomly sampled from two plants per pot. They were first fixed with 2.5% (v/v) glutaraldehyde in 0.1 M phosphate buffer solution (pH 7.4) under vacuum for 2–3 h at 20°C, and then post-fixed with 1% (w/v) osmium tetroxide in the phosphate buffer solution for 30 min [38]. Afterwards, they were dehydrated through a graded series of ethanol [50, 70, 80, 90 and 100% (v/v)], dried by a critical-point drying method with liquid CO_2_ and coated with metal and then loaded onto the instrument [38]. The surface scan was performed using a scanning electron microscope (FEI QUANTA200, Japan).

### Transmission electron microscopy

Squares were excised with scissors from the top-second leaf at the anthesis stage. The leaf samples of rice plants grown without slag (control) or with slag (H) applied at a rate of 935 mg plant-available Si per kg soil were collected and fixed immediately with 2% (v/v) glutaraldehyde and 2% (v/v) paraformaldehyde in 0.05 M sodium cacodylate buffer (pH 7.2) at room temperature overnight and then washed with the same buffer three times for 10 min each [38]. Afterwards, samples were postfixed with 1% (w/v) osmium tetroxide in the same buffer at room temperature for 2 h and washed twice with distilled water. The post-fixed samples were stained with 0.5% (w/v) uranyl acetate at 4°C overnight. They were then dehydrated in a graded series of ethanol [30, 50, 70, 80, 95, and 100% (v/v)] and three times in 100% ethanol for 10 min each [38]. Ultrathin sections (approximately 50 nm in thickness) were made with a diamond knife by an ultramicrotome (LKBVI). The sections were mounted on copper grids and stained for 7 min each with 2% (w/v) uranyl acetate and Reynolds' lead citrate [38]. The sections were examined by transmission electron microscopy (Phillips EM 400 ST, the Netherlands).

### Chemical analysis

The main chemical components of slag fertilizers were measured by SEM. Scanning electron microscopy was performed in a JSM-6510 SEM at accelerating voltage of 20 kV attached with an X-ray energy-dispersive spectrometer, EDS (Genesis XM2). Before the scanning process, all samples were dried and coated with gold to enhance the electron conductivity.

The available Si content in slag was determined following extraction with 0.5 M HCl [slag/(HCl) ratio of 1∶50, shaking at 300 rpm for 1 h] and analyzed by the colorimetric silicon molybdenum blue method [39]. Slag pH and EC were measured at a water/soil ratio of 2.5.

Plant-available Si content in soil was extracted by 0.25 M citric acid [soil/(citric acid) ratio of 1∶5] for 5 hrs, and analyzed by the colorimetric silicon molybdenum blue method [40]. The soil pH was measured at a water/soil ratio of 2.5.

The silicon content in rice plants was determined by the colorimetric silicon molybdenum blue method [41]–[42]. Briefly, 100 mg of plant tissue was mixed with 3 mL of 50% (w/v) NaOH in a polyethylene tube. These tubes were covered with loose-fitting plastic caps and autoclaved at 125°C for 1 h and analyzed by the colorimetric silicon molybdenum blue method.

### Statistical analysis

All data in figures and tables are shown as means ± SD of three replicates. Two -way ANOVA was used for statistical analysis and Fisher's L.S.D. test was adopted to detect the significant difference (*p*≤0.05) between the means of different treatments. All statistical analyses were done using the Excel 2007 and SPSS (PASW Statistics 18.0).

## Results

### Dry weight and silicon concentration of different rice tissues

Table 2–3 show that application of both iron slag (Q) and steel slag (H) fertilizers significantly increased dry weight of leaf and stem, and grain yield compared with the control (Si_0_) treatment. However, there was no significant difference among different application rates of silicon (except Si_0_). Dry weight of leaf and stem showed no significant difference between the two Si fertilizers tested, but Si fertilizer H produced significantly more grain weight than Si fertilizer Q.

**Table 2:** Effects of different silicon treatments on dry weight of rice organs (%).

Fertilizer	Rate	Leaf	Stem	Grain
Control	Si_0_	7.72±1.60 b	13.6±4.73 b	4.71±1.52 c
Q	Si_1_	13.1±2.02 a	20.8±1.11 a	13.7±0.69 b
	Si_2_	13.7±2.02 a	20.8±1.07 a	13.4±2.37 b
	Si_3_	13.0±2.27 a	20.2±1.46 a	11.8±1.99 b
H	Si_1_	12.8±2.71 a	19.4±1.30 b	15.1±2.33 ab
	Si_2_	11.5±1.08 a	20.2±1.24 a	15.0±1.53 ab
	Si_3_	12.5±1.15 a	23.7±2.40 a	16.9±2.08 a

Si_0_: no Si fertilizer; Si_1_: slag fertilizer applied at a rate of 187 mg plant-available Si per kg soil; Si_2_: slag fertilizer applied at a rate of 560 mg plant-available Si per kg soil; Si_3_: slag fertilizer applied at a rate of 935 mg plant-available Si per kg soil; H: slag fertilizer H, Q: slag fertilizer Q; Data are means ± SD of three replicates; mean values followed by different letters (a, b, c) are significantly different (*P*≤0.05).

**Table 3:** Analysis of variance of the effects slag-based silicon fertilizer (slag) and application rate of Si (Si-R) on dry weight of rice organs (%).

		F values		
Sources of variation	Df	Leaf	Stem	Grain
Slag	1	0.816 ns	0.142 ns	6.41*
Si-R	3	9.93*	11.61*	36.30**
Slag×Si-R	3	0.388 ns	1.03 ns	1.90 ns

Levels of probability: ns  =  non significant, significantly different *p≤0.05 and **p≤0.01.

Levels of probability: ns  =  non significant and *p≤0.05, **p≤0.01.

Table 4–5 show that the Si concentration was significantly different among different organs, with the order of leaf > stem > grain. Application of both Si fertilizers significantly increased the Si concentration in leaf, stem and grain compared with the Si_0_ treatment. The Si concentration in rice organs tended to increase with increasing application rate of Si, and there was a significant difference between the Si_3_ treatment and Si_1_ treatment. The Si concentration of stem was significantly higher in Si fertilizer H than in Si fertilizer Q. However, no significant difference in leaf or grain Si concentration was noted between the two Si fertilizers used.

**Table 4:** Effects of different silicon (Si) treatments on Si concentrations in rice organs (SiO_2_%).

Fertilizer	Rate	Leaf	Stem	Grain
Control	Si_0_	11.1±0.31 c	5.17±0.42 d	0.19±0.031 c
Q	Si_1_	12.3±0.36 b	5.57±0.47 cd	0.22±0.04 bc
	Si_2_	12.3±0.35 b	5.34±0.27 cd	0.23±0.04 bc
	Si_3_	12.1±0.31 b	5.76±0.14 c	0.27±0.02 ab
H	Si_1_	11.9±0.22 b	5.54±0.30 cd	0.21±0.03 c
	Si_2_	12.6±0.13 ab	6.31±0.39 b	0.26±0.02 ab
	Si_3_	12.9±0.27 a	6.87±0.37 a	0.28±0.04 a

Si_0_: no Si fertilizer; Si_1_: slag fertilizer applied at a rate of 187 mg plant-available Si per kg soil; Si_2_: slag fertilizer applied at a rate of 560 mg plant-available Si per kg soil; Si_3_: slag fertilizer applied at a rate of 935 mg plant-available Si per kg soil; H: slag fertilizer H, Q: slag fertilizer Q; Data are means ± SD of three replicates; mean values followed by different letters (a, b, c) are significantly different (*P*≤0.05).

**Table 5:** Analysis of variance of the effects of slag-based silicon fertilizer (slag) and application rate of Si (Si-R) on Si concentrations in rice organs (SiO_2_%).

		F values		
Sources of variation	Df	Leaf	Stem	Grain
Slag	1	2.09 ns	21.05**	0.602 ns
Si-R	3	30.93**	18.51**	13.74*
Slag×Si-R	3	1.80 ns	7.56*	0.786 ns

Levels of probability: ns  =  non significant, significantly different *p≤0.05 and **p≤0.01

### Disease severity

Under greenhouse conditions, rice leaves were naturally infected with brown spot caused by *Bipolaris oryzae*. At the anthesis stage, disease severity showed visible differences among treatments. The data in Table 6–7 demonstrate that rice leaf lesion of the control treatment (Si_0_) was most severe with an incidence of 39.6%, and a disease index of 56.0%. Application of both Si fertilizers significantly decreased brown spot development. Meanwhile, disease severity of fertilizer H treatments was lower than that of fertilizer Q treatments.

**Table 6:** Effects of different silicon (Si) treatments on rice brown spot development at the anthesis stage (%).

Fertilizer	Rate	Incidence of disease	Disease index
Control	Si_0_	39.7±2.11a	56.1±2.60 a
Q	Si_1_	4.67±0.60 b	22.0±3.50 b
	Si_2_	1.33±1.53 b	8.64±2.71bc
	Si_3_	0.33±0.58 b	2.22±3.85 c
H	Si_1_	3.67±2.08 b	18.0±2.26 bc
	Si_2_	1.33± 0.58 b	8.89±2.67bc
	Si_3_	0.00±0.0 b	0.00±0.00 c

Si_0_: no Si fertilizer; Si_1_: slag fertilizer applied at a rate of 187 mg plant-available Si per kg soil; Si_2_: slag fertilizer applied at a rate of 560 mg plant-available Si per kg soil; Si_3_: slag fertilizer applied at a rate of 935 mg plant-available Si per kg soil; H: slag fertilizer H, Q: slag fertilizer Q; Data are means ± SD of three replicates; mean values followed by different letters (a, b, c) are significantly different (*P*≤0.05).

**Table 7:** Analysis of variance of the effects of slag-based silicon fertilizer (slag) and application rate of Si (Si-R) on rice brown spot development at the anthesis stage (%).

		F values	
Sources of variation	Df	Incidence of disease	Disease index
Slag	1	0.0145 ns	13.20*
Si-R	3	47.05**	3545**
Slag×Si-R	3	0.00727 ns	5.85 ns

Levels of probability: ns  =  non significant, significantly different *p≤0.05 and **p≤0.01.

### Transmission electron microscopic analysis of silica cell

**Figure 1:**
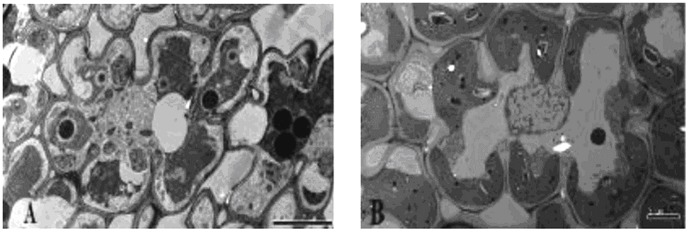
Transmission electron micrographs of mesophyll cells of rice leaves. Scale bars = 5 µm. **A**: Mesophyll cells of a control plant grown without silicon fertilizer at the anthesis stage; **B**: Mesophyll cells of a silicon-treated plant grown with slag (H) applied at a rate of 935 mg plant-available Si per kg soil at the anthesis stage.

**Figure 2:**
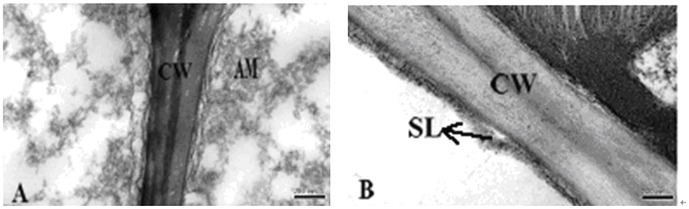
Transmission electron micrographs of cell wall from leaves of rice. CW, cell wall; AM, amorphous material; SL, silicon layer. A: Leaf epidermis of a control plant grown without silicon fertilizer at the anthesis stage; B: Leaf epidermis of a silicon treated plant with slag (H) applied at a rate of 935 mg plant-available Si per kg soil at the anthesis stage.

**Figure 3:**
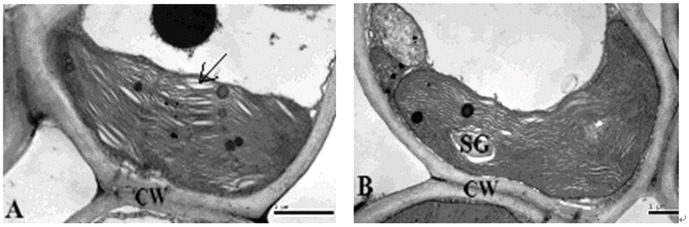
Transmission electron micrographs of chloroplasts from leaves of rice. Scale bars  = 1 µm. CW, cell wall; SG, starch grain. **A**: Chloroplast of a control plant grown without silicon fertilizer at the anthesis stage; **B**: Chloroplast of a silicon treated plant grown with slag (H) applied at a rate of 935 mg plant-available Si per kg soil at the anthesis stage.

Ultrathin sections of leaf samples were observed by transmission electron microscope (TEM). The ultrastructural details demonstrated that the numbers of fungal cells and fungal colonization in the leaf epidermis were different between Si-untreated and Si-treated rice plants. The leaf mesophyll cells of silicon-untreated plants were disorganized at the stage of the fungal (*Bipolaris oryzae*) colonization. The cytoplasm was disintegrated with a consequence of chloroplast degradation and cell-wall alterations. Abundant amorphous materials were noticed in a mesophyll cell colonized by the fungus (Figure 1). The Si layers were observed in Si-treated epidermal cell walls, and the thickness of the silicon layer was seen to be increased by Si application (Figure 2). The chloroplast thylakoid lamella of mesophyll cells of Si-untreated leaves became swollen, and stroma lamellae and grana lamellae of chloroplasts were distorted. In contrast, the chloroplast structure of mesophyll cells of Si-treated rice leaves was relatively intact, with thylakoid lamellae stacked in order, grana lamellae accumulated compactly and some starch grains visible (Figure 3).

### Scanning electron microscopic analysis of silica cells

**Figure 4:**
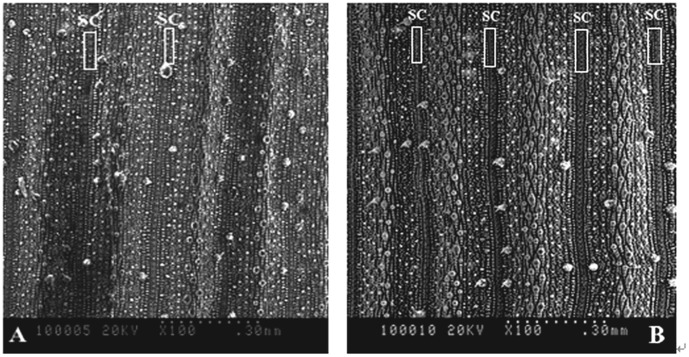
Scanning electron micrographs with 100 magnification of rice leaves. Scale bars  = 30 mm. SC, silica cell. **A**: the top second rice leaf epidermis of a control plant without silicon fertilizer at the anthesis stage; **B**: the top second rice leaf epidermis of a silicon-treated rice plant grown with slag (H) applied at a rate of 935 mg plant-available Si per kg soil at the anthesis stage.

**Figure 5:**
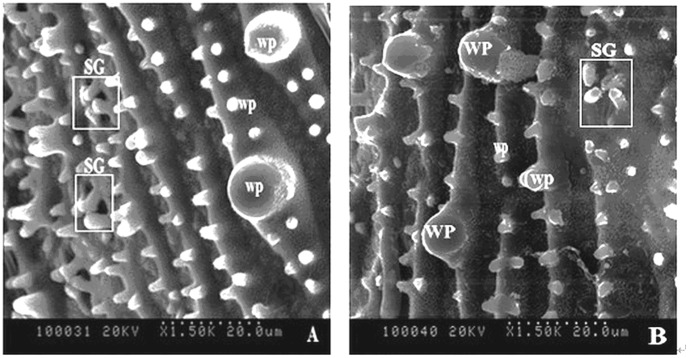
Scanning electron micrographs with 150 K magnification of rice leaves. Scale bars  = 20 µm. SC, silica cell; WP, wart-like protuberance; SG, stomatal guard cell. **A**: **t**he top second leaf epidermis of a control plant without silicon fertilizer at the anthesis stage; **B**: The top second leaf epidermis of a silicon-treated rice plant grown with slag (H) applied at a rate of 935 mg plant-available Si per kg soil at the anthesis stage.

Morphology of silica cells on the surface of the top-second leaf at the anthesis stage differed among treatments (Figure 4, 5). There were many silica cells, wart-like protuberances (papillae) and stomata on the leaf surface. The silica cells had a dumbbell shape and were distributed in rows along the leaf veins. However, the morphology and number of these silica cells varied among treatments. Silicon application led to more pronounced cell silicification in rice leaves, more silica cells and larger papillae.

## Discussion

In this study, the concentration of plant-available Si in the soil tested was 41.8 mg (Si) kg^−1^. In China, the critical value for plant-available Si concentration in acid paddy soil is 44.4–51.4 mg kg^−1^ (Si), below which positive rice responses to silicate fertilizer can be expected [29]. Our results show that silicon fertilizers from steel slag and iron slag both significantly promoted rice growth and rice yield. Silicon fertilizer H produced significantly higher grain weight than silicon fertilizer Q at the same plant-available Si application rate. Two factors may account for this observation. First, the composition and cooling process of slags influence Si-dissolution from slags. Slag H, which was cooled slowly, had higher Si-availability to plants compared with slag Q, which was more rapidly cooled in water. This result was consistent with a report by Takahashi (1981) [43], suggesting that Si availability of slag to plants cannot be precisely determined only by the extraction method using 0.5 M HCl. It is necessary to estimate the Si-releasing process from slags in paddy soils and to analyze the factors affecting the solubility of the slags in future studies. Second, other nutrients provided by slag might be also beneficial for rice growth, such as Ca, Mg, Fe and Mn etc. In this study, the plant-available Si concentration was lower in slag H than in slag Q, thus, at the same available-Si application rate, the real application rate of the slag H was higher than that of slag Q. In this case, the amount of other nutrients such as Ca, Fe and Mn provided by slag H might be higher than that by slag Q because not only the real application rate of slag H was higher than that of slag Q but also the content of Ca, Fe and Mn was higher in slag H than in slag Q (Table 1). It could be supposed that other nutrients provided by slags also contributed to the final rice performance, dry weight and rice yield, which, however, needs further validation.

Silicon fertilizer could be an environmentally-friendly alternative to control rice diseases [14], [44]–[46]. In this study, rice leaves were naturally infected with brown spot disease caused by *Bipolaris oryzae* at the jointing stage. The leaves of rice plants that were not treated with slag showed disease symptoms 5 days earlier than those treated with slag. At anthesis, visible differences in disease severity appeared among treatments. We found that application of both steel slag and iron slag fertilizers showed significantly lower brown spot incidence and severity (Table 6–7). Lesion areas of leaves showed a decreasing, but non-significant trend with increasing Si application rates (Table 4–5). This result was consistent with rice yield (Table 2). The ultrastructural characteristics showed that the chloroplast thylakoid lamellae of mesophyll cells of untreated rice leaves became swollen, and stroma and grana lamellae of chloroplast were distorted at anthesis. However, the chloroplast structure of mesophyll cells of Si-treated leaves was relatively intact (Figure 3).

Brown spot severity has been reported to be negatively correlated with Si concentration in rice tissue [15], [24], [47]. An active Si uptake by lateral roots of rice plants plays a key role in rice resistance to brown spot [24]. Application of the two Si fertilizers significantly increased the Si concentration in leaves (Table 2). There have been debates of the mechanisms involved in Si-mediated plant disease resistance. Some authors suggest that a mechanical or physical barrier provided by Si deposition in cell walls contributes to enhanced resistance [38], [48]–[49], while more recent studies suggest that Si plays a biochemical role in mediating plant resistance to pathogens [33], [46], [50]. Our results show that Si application led to more pronounced cell silicification in rice leaves and more elaborate and larger papillae (Figure 4, 5). The elaborate papillae formed in Si-treated leaf epidermal surface might increase the resistance to fungal penetration [37], [51]. The Si layers were observed in Si-treated epidermal cell walls, and their thickness was increased by Si treatment (Figure 2). The Si layers in epidermal cell walls supposedly confer enhanced host resistance to brown spot, which is in line with the previous reports that the cuticular Si double layer developed on rice leaf cells constituted a physical barrier to impede fungal penetration and colonization [38], [48]–[49].

In this study, we also found apparent differences in the number of fungal cells and fungal colonization in the leaf epidermis between Si-untreated and Si-treated plants (Figure 1). We surmise that soluble Si may induce physiological resistance to restrain the growth of *Bipolaris oryzae* and keep host cells relatively intact. Rodrigues et al. (2003a, 2005b) suggested that Si induced accumulation of phenolic compounds or phytoalexins, which played a primary role in rice defense against infection by *Magnaporthe grisea* [31], [52]. Dallagnol et al. (2011) found that the concentrations of soluble phenolics and lignin and activities of peroxidase and chitinase were higher in Si-treated rice leaves infected by *Bipolaris oryzae*, which contributed to rice resistance to brown spot [47]. Other reports suggest that after inoculation with *M. grisea*, Si-treated rice plants significantly increased the activities of pathogenesis-related proteins (PRs) in leaves, such as peroxidase (POD), polyphenol oxidase (PPO), phenylalanine ammonia lyase (PAL), and catalase (CAT) [37], [46]. Therefore, we believe that Si-enhanced plant disease resistance plus the role of Si as physical barrier as suggested by Sun et al. (2010) in rice blast resistance [46] also contributed to the Si-enhanced resistance to rice brown spot observed in the present study.

### Conclusions

Applying Si fertilizer to Si-deficient paddy soil is necessary for both high rice yield and brown spot resistance. Both steel slag and iron slag are effective in this regard. In this experiment, silicon fertilizer H produced significantly higher grain weight than silicon fertilizer Q at the same plant-available Si application rate. Composition and cooling process of slags influence Si-dissolution from slags. Si availability of slag to plants cannot be precisely determined only by the extraction method using 0.5 M HCl. The immobile silicon deposited in host cell walls and papillae sites is the first physical barrier for fungal (*Bipolaris oryzae*) penetration and soluble Si in the cytoplasm enhances physiological or induced resistance to restrain fungal colonization.

## References

[1] Epstein E (1994) The anomaly of silicon in plant biology. Proc Natl Acad Sci USA 91: 11–17. 11607449 10.1073/pnas.91.1.11 PMC42876

[2] Ma JF, Takahashi E (2002) Soil, Fertilizer, and Plant Silicon Research in Japan. Elsevier, Amsterdam, pp. 1–2.

[3] Epstein E (1999) Silicon. Ann Rev Plant Physiol Plant Mol Biol 50: 641–664. 15012222 10.1146/annurev.arplant.50.1.641

[4] Ma JF (2004) Role of silicon in enhancing the resistance of plants to biotic and abiotic stresses. Soil Sci Plant Nutr 50: 11–18.

[5] Rodrigues FÁ, Datnoff LE (2005a) Silicon and rice disease management. Fitopatol Bras 30: 457–469.

[6] Liang YC, Sun WC, Zhu YG, Christie P (2007) Mechanisms of silicon-mediated alleviation of abiotic stresses in higher plants: A review. Environ Pollut 147: 422–428. 16996179 10.1016/j.envpol.2006.06.008

[7] Catherine Keller FG, Meunier JD (2012) Benefits of plant silicon for crops: a review. Agron Sust Develop 32: 201–213.

[8] Wailes EJ, Cramer GL, Chavez EC, Hansen JM (1997) Arkansas global rice model: international baseline projections for 1997–2010. Arkansas Agric Exp Stat, Arkansas. pp. 1–46.

[9] van Nguyen N, Ferrero A (2006) Meeting the challenges of global rice production. Paddy Water Environ 4: 1–9.

[10] Ma JF, Yamaji N (2006) Silicon uptake and accumulation in higher plants. Trends Plant Sci 11: 392–397. 16839801 10.1016/j.tplants.2006.06.007

[11] Raven JA (2003) Cycling silicon–the role of accumulation in plants. New Phytol 158: 419–430. 10.1046/j.1469-8137.2003.00778.x 36056512

[12] Foy CD (1992) Soil chemical factors limiting plant root growth. Adv Soil Sci 19: 97–149.

[13] Winslow MD, Okada K, Correa-Victoria F (1997) Silicon deficiency and the adaptation of tropical rice ecotypes. Plant Soil 188: 239–248.

[14] Datnoff LE, Deren CW, Snyder GH (1997) Silicon fertilization for disease management of rice in Florida. Crop Prot 16: 525–531.

[15] Deren CW, Datnoff LE, Snyder GH, Martin FG (1994) Silicon concentration, disease response, and yield components of rice genotypes grown on flooded organic histosols. Crop Sci 34: 733–737.

[16] Savant NK, Snyder GH, Datnoff LE (1996) Silicon management and Sustainable rice production. Adv Agron 58: 151–199.

[17] Alvarez J, Datnoff LE (2001) The economic potential of silicon for integrated management and sustainable rice production. Crop Prot 20: 43–48.

[18] Bocharnikova EA, Loginov SV, Matychenkov VV, Storozhenko PA (2010) Silicon fertilizer efficiency. Russ Agric Sci 36: 446–448.

[19] Ou SH (1985) Rice diseases, 2nd ed. Kew, Surrey, UK, Commonwealth Mycological Institute.

[20] Motlagh MR, Kaviani B (2008) Characterization of new bipolaris spp.: the causal agent of rice brown spot disease in the North of Iran. Int J Agric Biol 10: 638–642.

[21] Marchetti MA, Peterson HD (1984) The role of *Bipolaris oryzae* in floral abortion and kernel discoloration in rice. Plant Dis 68: 288–291.

[22] Lee TS, Hsu LS, Wang CC, Jeng YH (1981) Amelioration of soil fertility for reducing brown spot incidence in the patty field of Taiwan. J Agric Res Chin 30: 35–49.

[23] Datnoff LE, Snyder GH, Raid RN, Jones DB (1991) Effect of calcium silicate on blast and brown spot intensities and yields of rice. Plant Dis 75: 729–732.

[24] Dallagnol LJ, Rodrigues FÁ, Mielli MVB, Ma JF, Datnoff LE (2009) Defective active silicon uptake affects some components of rice resistance to brown spot. Phytopathol 99: 116–121. 10.1094/PHYTO-99-1-0116 19055443

[25] Wu SP, Xue YJ, Ye QS, Chen YC (2007) Utilization of steel slag as aggregates for stone mastic asphalt (SMA) mixtures. Build Environ 42: 2580–2585.

[26] Motz H, Geiseler J (2001) Products of steel slags an opportunity to save natural resources. Waste Manage 21: 285–293. 10.1016/s0956-053x(00)00102-1 11280521

[27] Shen HT, Forssberg E (2003) An overview of recovery of metals from slags. Waste Manage 23: 933–949. 10.1016/S0956-053X(02)00164-2 14614927

[28] Zhu GL (2010) The current state and developing of comprehensive disposal of steel and iron slag. Iron Steel Scrap 1: 12–16 (in Chinese)..

[29] Wang HL, Li CH, Liang YC (2001) Agricultural utilization of silicon in China. In: Silicon in Agriculture. Datnoff LE, Snyder GH, Korndorfer GH. ed. Elsevier, Amsterdam, pp. 343–358.

[30] Seebold KW, Kucharek TA, Datnoff LE, Correa Victoria FJ, Marchetti MA (2001) The influence of silicon on components of resistance to blast in susceptible, partially resistant, and resistant cultivars of rice. Phytopathol 91: 63–69. 10.1094/PHYTO.2001.91.1.63 18944279

[31] Rodrigues FÁ, Benhamou N, Datnoff LE, Jones JB, Bélanger RR (2003a) Ultrastructural and cytochemical aspects of silicon-mediated rice blast resistance. Phytopathol 93: 535–546. 10.1094/PHYTO.2003.93.5.535 18942975

[32] Rodrigues FÁ, Valeb FXR, Korndörfer GH, Prabhud AS, Datnoff LE, et al (2003b) Influence of silicon on sheath blight of rice in Brazil. Crop Prot 22: 23–29.

[33] Rodrigues FÁ, Vale FXR, Datnoff LE, Prabhu AS, Korndörfer GH (2003c) Effect of rice growth stages and silicon on sheath blight development. Phytopathol 93: 256–261. 10.1094/PHYTO.2003.93.3.256 18944334

[34] Rodrigues FÁ, McNally DJ, Datnoff LE, Jones JB, Labbé C, et al (2004) Silicon enhances the accumulation of diterpenoid phytoalexins in rice: A potential mechanism for blast resistance. Phytopathol 94: 177–183. 10.1094/PHYTO.2004.94.2.177 18943541

[35] Cha W, Kim J, Choi H (2006) Evaluation of steel slag for organic and inorganic removals in soil aquifer treatment. Water Res 40: 1034–1042. 16490232 10.1016/j.watres.2005.12.039

[36] Naoto K, Naoto O (1997) Dissolution of Slag Fertilizers in a Paddy Soil and Si Uptake by Rice Plant. Soil Sci Plant Nutr43: 329–341.

[37] Cai KZ, Gao D, Luo SM, Zeng RS, Yang JY, et al (2008) Physiological and cytological mechanisms of silicon-induced resistance in rice against blast disease. Physiol Plant 134: 324–333. 18513376 10.1111/j.1399-3054.2008.01140.x

[38] Kim SG, Kim KW, Park EW, Choi D (2002) Silicon-induced cell wall fortification of rice leaves: A possible cellular mechanism of enhanced host resistance to blast. Phytopathol 92: 1095–1103. 10.1094/PHYTO.2002.92.10.1095 18944220

[39] Buck GB, Korndörfer GH, Datnoff LE (2011) Extractors for estimating plant available silicon from potential silicon fertilizer sources. J Plant Nutr 34: 272–282.

[40] Lu RK (2000) Analytical methods for soil and agro-chemistry. Chinese Agricultural Technology, Beijing, pp. 201–203 (in Chinese).

[41] Nanayakkara UN, Uddin W, Datnoff LE (2008) Effects of soil type, source of silicon, and rate of silicon source on development of gray leaf spot of perennial ryegrass turf. Plant Dis 92: 870–877. 10.1094/PDIS-92-6-0870 30769716

[42] Dai WM, Zhang KQ, Duan BW, Sun CX, Zheng KL, et al (2005) Rapid determination of silicon content in rice (Oryza sativa). Chinese J Rice Sci 19: 460–462 (in Chinese)..

[43] Takahashi K (1981) Effects of slags on the growth and the silicon uptake by rice plants and the available silicates in paddy soils. Bulletin Shikoku Agric Exp Stat 38: 75–114.

[44] Liang YC, Ma TS, Li FJ, Feng YJ (1994) Silicon availability and response of rice and wheat to silicon in calcareous soils. Commun Soil Sci Plant Anal 25(13& 14): 2285–2297.

[45] Seebold KW, Datnoff LE, Correa-Victoria FJ, Kucharek TA, Snyder GH (2004) Effects of silicon and fungicides on the control of leaf and neck blast in upland rice. Plant Dis 88: 253–258. 10.1094/PDIS.2004.88.3.253 30812356

[46] Sun WC, Zhang J, Fan QH, Xue GF, Li ZL, et al (2010) Silicon-enhanced resistance to rice blast is attributed to silicon-mediated defence resistance and its role as physical barrier. Eur J Plant Pathol 128: 39–49.

[47] Dallagnol LJ, Rodrigues FÁ, DaMatta FM, Mielli M B, Pereira SC (2011) Deficiency in silicon uptake affects cytological, physiological, and biochemical events in the rice–*Bipolaris oryzae* interaction. Phytopathol 101: 92–104. 10.1094/PHYTO-04-10-0105 20879842

[48] Yoshida S (1965) Chemical aspects of the role of silicon in physiology of the rice plant. Bull Shikoku Agr Exp Stat 15: 1–58.

[49] Hayasaka T, Fujii H, Ishiguro K (2008) The role of silicon in preventing appressorial penetration by the rice blast fungus. Phytopathol 98: 1038–1044. 10.1094/PHYTO-98-9-1038 18943742

[50] Liang YC, Sun WC, Si J, Römheld V (2005) Effect of foliar- and root-applied silicon on the enhancement of induced resistance in *Cucumis sativus* to powdery mildew. Plant Pathol 54: 678–685.

[51] Zhang GL, Gen DQ, Zhang HC (2006) Silicon Application enhances resistance to sheath blight (*rhizoctoniasolani*) in rice. J Plant Physiol Mol 32: 600–606 (in Chinese).. 17075186

[52] Rodrigues FÁ, Jurick WM, Datnoff LE, Jones JB, Rollins JA (2005b) Silicon influences cytological and molecular events in compatible rice-*Magnaporthe grisea* interactions. Physiol Mol Plant Pathol 66: 144–159.

